# Advance care planning and advance directives: an overview of the main critical issues

**DOI:** 10.1007/s40520-021-02001-y

**Published:** 2021-10-15

**Authors:** Cristina Sedini, Martina Biotto, Lorenza M. Crespi Bel’skij, Roberto Ercole Moroni Grandini, Matteo Cesari

**Affiliations:** 1 Department of Clinical Sciences and Community Health, University of Milan, Milan, Italy; 2 Hospice Cascina Brandezzata, IRCCS Ca’ Granda-Ospedale Maggiore Policlinico, Milan, Italy; 3 Geriatric Unit, IRCCS Istituti Clinici Scientifici Maugeri, Milan, Italy

**Keywords:** Palliative care, End of life, Geriatrics, Aging, Decision-making capacity, Functional limitations

## Abstract

Advance care planning (ACP) is a process that guarantees the respect of the patient’s values and priorities about his/her future care at the end of life. It consists of multiple conversations with the health professional that may lead to the completion of Advance Directives (AD), a set of legal documents helpful to clinicians and family members for making critical decisions on behalf of the patient, whereas he/she might become incapable. Over the past years, ACP has become particularly relevant for the growth of chronic diseases, the increase in life expectancy, and the growing attention paid to the patient’s decisional autonomy. Several nations have introduced specific regulations of ACP and AD. However, their diffusion is accompanied by unforeseen limitations and issues, burdening their complete and systematic adoption. The present article describes several controversial aspects of ACP and some of the most significant challenges in end-of-life care.

## Introduction

Advance care planning (ACP) is a process designed to help the patient (from the onset of disease till the end of life) at defining the future medical and end-of-life care interventions according to his/her values, wishes, and preferences [1]. ACP implies clear communication between the person, his/her family, and the medical staff that will take care of him/her. Although it is not the primary objective of the process, ACP can lead to the drafting of the so-called Advance Directives (AD). In these written documents, the individual expresses his/her personal preferences about future medical treatments in case he/she might become incapacitated to present them [2]. However, an individual can write AD alone at any time of his life, even in the absence of illness.

The worldwide increase of chronic diseases, the increasing life expectancy, and the importance of personalizing care have significantly raised interest and discussions around the ACP. In these recent years, many countries have been developing specific laws to regulate and improve the quality of care offered at the end of life. Nevertheless, many weaknesses and critical issues frequently (and not surprisingly) arise, given the delicate matter involving relevant ethical aspects. In the present article, we overview the most significant points discussed in the literature and provide examples of how some countries have tried to address them.

## Informed consent

The core of health and social care is to respect the autonomy of the person who has the right to accept or refuse the proposed interventions. The informed consent is widely considered as a form of respect for the individual’s autonomy. However, it is valid only if the person is capable, adequately informed, and not coerced [3].

On a practical level, seeking a person’s informed consent could be challenging, especially when the individual has lost the ability to understand relevant medical information and critically discriminate among alternatives. This is a pretty common issue in geriatric medicine, where older people often present frailty, chronic conditions, and/or functional declines affecting their decision-making capacity [4]. AD may, thus, represent an ideal instrument for properly planning medical care according to the person’s wishes when he/she is still able to describe them [5]. The timely completion of AD is critical and requires that the person’s values and preferences are discussed and recorded per time. Interestingly, evidence suggests a possible positive impact of AD on the management of persons with degenerative and/or terminal illnesses across care settings. For example, it has been demonstrated that AD are associated with a decreased risk of hospitalization, higher concordance between the individual’s end-of-life wishes with the provided care [6], reduction of unnecessary/invasive procedures (e.g., feeding tube insertion) [7], and amelioration of the caregiver’s stress [8].

## Person’s autonomy and ethics in medicine

In real life, it is often not easy to reconcile the decisions previously expressed by the person with the decisions the clinician may take based on his/her competence and ethical principles. Both the individual and the clinician may risk of sustaining life at all costs with disproportionate, unnecessary, or useless treatments. Similarly, they may refuse treatment proposals, refraining from the diagnostic–therapeutic process and relief of sufferance. This controversial point can be expressed by the so-called “binding force” concept, describing the clinician’s conflictual relationship with AD that he/she disagrees with.

Some clinicians may prioritize the individual’s autonomy in the decision-making process despite their personal beliefs and objections. On the other hand, some clinicians may refuse to follow the patient’s AD because of seeing a mistake in the non-administration of the intervention for a potentially reversible condition based on scientific evidence. Furthermore, it could be possible that the clinician may consider irrelevant the request included in the AD when the intervention would not make the person’s interest (e.g., adoption of invasive treatments in the presence of an end-stage disease) [9]*.* It has also been reported of patients receiving life-extending treatments in their last days of life, despite having previously expressed their preference for only symptoms control [10].

In emergency settings, the importance of acting with prompt interventions makes AD an obstacle for clinicians because of the lack of time to find, read, and interpret the documentation. For this reason, aggressive treatments are often administered at the Emergency Departments without adequately considering the individual’s will [9, 11]. It is also noteworthy that some clinicians believe that people lack the required medical knowledge for properly judging the pros and cons of interventions. Another obstacle to applying AD is sometimes represented by the family opposition to the person’s decisions [12]. If a family member requests a deviation from the person’s AD, the clinician may find him/herself trying to mediate, subjectively defining solutions.

## How to inform a person about ACP

Usually, the healthcare systems are not designed to systematically and properly approach the ACP. To date, there are no updated guidelines to guide this process to appropriate person-centered results [13]. However, some general recommendations [14–17] explain that the ACP discussion should be initiated by a formally trained person, who could be a healthcare professional (e.g., a clinician, a nurse, or a psychologist), a social worker, or a lawyer. It is not always clear who should help the person with (1) the drafting of the ACP, and (2) the collection of eventual future changes. A professional guide with appropriate knowledge of diseases, prognosis, and possible treatments is needed to complete the AD in a clinically relevant, feasible, and optimally informed way.

Informing the person is, of course, at the very base of a shared and agreed intervention plan. In fact, it is recommended a team approach, where the clinicians and the care team work together in support of the person by providing explanations, discussions, and counseling about ACP according to each one’s specificity and experience [8, 13].

## Timing and settings for the ACP and AD

Although there are no precise indications about the "perfect timing" to write the AD, there are some suggestions. It surely depends on whether the person is healthy, has mild-to-moderate chronic diseases, or presents an advanced life-threatening illness with the risk of imminent death. A medical crisis or a recent hospital admission may be identified as occasions to think about future healthcare decisions but do not represent the best moment for this kind of discussion. In fact, decisions made in the presence of a potentially reversible acute condition (e.g., delirium) may affect the individual’s decision-making capacity, resulting in untrustworthy and inaccurate AD [18]. Furthermore, Enguidanos and Ailshire [19] found that AD completed in the last three months of life are associated with a higher likelihood of aggressive care preferences in a cohort study of US adults. The authors hypothesized this might result from hurried discussions conducted during urgent procedures, the pressure coming from the healthcare system, and/or the individual’s fear of dying. Differently, persons who prepared their AD one year or more before death were more likely to prefer limited/conservative care.

Imagining future diseases and incapacities may be challenging for healthy young adults, potentially affecting the accuracy of the expressed preferences. Indeed, it is recommended to regularly review the AD, particularly every time a change in the individual’s health status and/or values change [17].

Regardless of age, it is not recommended to postpone the decision to discuss ACP and AD in some clinical situations. For example, when a person is at risk of losing his/her mental capacities because of degenerative diseases (e.g., dementia).

Ideally, ACP should be offered during routine clinical practice before the individual becomes acutely unwell. Some authors suggest initiating the ACP in primary care or the outpatient setting [20]. The General Practitioner (GP) is indeed the healthcare professional who could better than others initiate the discussion about ACP with the person because in the position of best knowing his/her clinical conditions and potentially having followed him/her over time. The GP should actively encourage the individual to consider the ACP if his/her clinical (in particular, mental) conditions worsen [21]. The GP should regularly offer ACP guidance, document all the relevant discussions, periodically review the existing ACP, assess the current mental capacities to make advance decisions, and eventually involve other specialists to inform the person better [22]. At the same time, some issues are undoubtedly present in this setting, such as time constraints and adequate training in the specific communication about life-sustaining medical treatments [23, 24].

Nursing homes also appear as an adequate setting because, once individuals are more settled, there is time to know them, meet their families, and discuss future medical choices. Nevertheless, many persons are admitted to a nursing home when they are alone, their abilities no longer allow them to remain at home, and/or have cognitive impairment [24].

## Contents of AD

Questioning the information included in the AD means trying to understand how its content can affect its usefulness. So far, there are no international guidelines on the minimum content of the AD. Laws regulating the AD and terminology are highly heterogeneous across countries. For example, the AD are recognized in the United States as the Living Will (LW) and the Durable Power of Attorney for Health Care (DPAHC). In contrast, in the United Kingdom, AD are known as Advance Decision to Refuse Treatment (ADRT) or LW. Most countries have proposed how an AD should be completed, sometimes designing specific templates [25, 26]. In this attempt to standardize the methodology, no-profit organizations supporting human rights and end-of-life care have also been playing an important role [27, 28]*.*

An AD may be very detailed but also very general, with consequent pros and cons. As discussed in the Council of Europe document [29], if the AD are “too precise, they leave no room for any medical interpretation with a view to adaptation, whereas if they are too general, they make it impossible to be certain that the wish expressed will have anything to do with the specific clinical situation”.

Another limit is when the drafting of the AD is completed before their eventual application. The person’s views might change over time, also according to his/her health status. It could also happen that possible changes in the person’s preferences cannot be communicated because of the worsening of his/her conditions, constituting a limit to overcome [9].

Most AD include information regarding the patient’s preferences for interventions (e.g., antibiotics, hydration, feeding, use of ventilators, cardiopulmonary resuscitation, analgesia), life-sustaining treatments, resuscitation, and a surrogate decision-maker.

Sometimes AD include vague or ambiguous instructions so that the physician should find confirmation by involving the person in the decision-making process if possible. If the person is incapable of clarifying, a surrogate decision-maker may provide support to the healthcare professional to better understand what the patient meant.

Although easier to standardize and disseminate, checkbox-based written directives may be insufficient for solving specific problems on how to provide the expected care. There is also the risk they could become a further bureaucratic complication in the relationship between the person and the clinician. For these limitations, multidisciplinary and multicomponent ACP interventions may be more effective at meeting the person’s preferences than the written legal documents alone [30].

## Surrogate decision-maker

The AD can include the “durable powers of attorney for health care” and the “health care proxy appointment”, which allow an individual to choose a surrogate decision-maker who takes care decisions on his/her behalf in the event of his/her incapacity. As previously discussed, not every decision can be clarified by the AD; for this reason, it is highly recommended to identify a care proxy.

Substitute decision-maker will need to be available and contactable, aged 18 years or older, and prepared to clearly and confidently advocated on behalf of the person when talking to clinicians. Regardless of the cultural background, most people prefer to choose the surrogate decision-maker among family members [31, 32]. Identifying a substitute decision-maker offers some advantages for the person and next of kin, including better end-of-life care and more satisfaction (of the person and his/her family) about the received care [8].

Nevertheless, some problems might also arise with the surrogate decision-makers. First, some studies have shown possible non-concordance between the persons’s preferences and those of the family members [33]. For example, it has been reported that relatives would choose more aggressive end-of-life treatments than the patient him/herself [34]. Sometimes, the clinician and the surrogate decision-maker are not in the position of following the instructions of a LW because the patient’s request is in contrast with the country’s laws. Or, other therapeutic options not foreseeable at the time of subscription of AD have become available. If decisions of the surrogate decision-maker are in contrast with the patient’s values and written LW, if next of kin is acting in his/her self-interest, and/or he/she does not agree with the medical care, the intervention of a legal guardian appointed by the court is possible in many countries. In these cases, the LW seems to be only a further bureaucratic complication and slows down supportive care.

## Shared information about ACP

It is difficult to share the preferences expressed by the patient with all the professionals who take care and will take care of him/her in different settings over time. The SOP model (Shared decision-making with Oncologists and Palliative care specialists), consisting in the integration of oncologists and palliative care professionals, is an example for the implementation of Do-Not-Resuscitate preferences in patients with advanced cancer, allowing the allocation of personalized care. The process of shared decision-making might indeed help patients receiving end-of-life care according to their preferences [35].

Another way to share the person’s end-of-life decisions is the use of national databases, which in some countries exist since many years. An example is the “US Advance Care Registry”, a database that contains all types of end-of-life documents and making them available to all clinicians on the web [36].

In Italy, after the approval of the law 217/2019 [37], a national database was established [38]. It collects the advance processing instructions stored by the municipalities and notaries. The database can be accessed by the individual, the trustee appointed by him, and the clinician who is treating the patient [39].

## Strategies and future prospectives

Although there are several limitations and critical issues, the ACP is associated with positive outcomes [40] and should be encouraged. At the basis of the ACP, there is the correct timing, the sharing of the medical information, the empathic discussion, and the final decision. AD should result from a multistep, multidisciplinary, and good quality discussion between the person, his/her family, and healthcare professionals. AD too focused on medical instructions are not always appropriate, while those including the person’s values better allow the medical team to interpret the person’s preferences [9].

Over the years, some innovations have been developed to assist the person with decision-making related to ACP. These are three types of tools: those used in face-to-face meetings, those designed for use outside of clinical meetings (e.g., take-home materials), and those that adopting instruments like telephone or video [41–43]. Although not all tools have been formally tested in research settings, some of them are already used, offering practical benefits. For example, PREPARE [44] is an ACP website with videos that introduces the individual to ACP and prepares him/her to decision-making. The Conversation Project [45] is a written tool kit with value-based questions helping individuals to start ACP conversations. Similarly, the “Making your Wishes Known” is a tool providing video instructions and explanations on how to complete AD [46]. These interventions help people to think, autonomously or in collaboration with his/her family, about the different options and consider the relevant aspects [41].

Information campaigns about AD through social media, televisions, radio, and newspapers are needed to reach all generations and ethnic minorities. Teaching bioethical competencies and communication skills to medical students is also important to improve and increase the adoption of ACP. The specialist who diagnoses a disease with a poor prognosis should be more ready at taking full charge of the patient, informing him/her about alternative strategies (e.g., palliative care), planning the long-term care path, and making sure that all the clinicians who will be involved in the future management of the case will be aware of the person’s preferences.

In conclusion, the relationship between the clinicians and the person should not be regulated by too rigid laws, but modulated within a therapeutic alliance. This is a relationship between a person in the need of help and a health professional who put him/herself in the perspective of offering better and individualized care.
